# Supplementary material for: Comparative multiomics analysis of cell physiological state after culture in a basket bioreactor
Source: Sci Rep. 2022 Nov 23;12:20161. doi: 10.1038/s41598-022-24687-4 (PMC9686226; doi:10.1038/s41598-022-24687-4)
Supplement: Supplementary file 1 — Supplementary Information 1. [file 41598_2022_24687_MOESM1_ESM.zip › raw data/Transcriptomic raw data/6.Enrichment/1.GO/TE1vsTC1/GSEA/TC1/EXCITATORY_EXTRACELLULAR_LIGAND_GATED_ION_CHANNEL_ACTIVITY/EXCITATORY_EXTRACELLULAR_LIGAND_GATED_ION_CHANNEL_ACTIVITY(GO_0005231).html]

Details for gene set EXCITATORY\_EXTRACELLULAR\_LIGAND\_GATED\_ION\_CHANNEL\_ACTIVITY(GO:0005231)[GSEA]


|  || Dataset | readcount.sample |
| Phenotype | sample.cls |
| Upregulated in class | TC1 |
| GeneSet | EXCITATORY\_EXTRACELLULAR\_LIGAND\_GATED\_ION\_CHANNEL\_ACTIVITY(GO:0005231) |
| Enrichment Score (ES) | -0.3523708 |
| Normalized Enrichment Score (NES) | -1.0 |
| Nominal p-value | 0.0 |
| FDR q-value | 1.0 |
| FWER p-Value | 1.0 |
Table: GSEA Results Summary

  

Fig 1: Enrichment plot: EXCITATORY\_EXTRACELLULAR\_LIGAND\_GATED\_ION\_CHANNEL\_ACTIVITY(GO:0005231)
  
Profile of the Running ES Score & Positions of GeneSet Members on the Rank Ordered List

  

| PROBE | DESCRIPTION   (from dataset) | GENE SYMBOL | GENE\_TITLE | RANK IN GENE LIST | RANK METRIC SCORE | RUNNING ES | CORE ENRICHMENT || 1 | 103218664 | GRIN2B |  |  | 6 | Infinity | 0.0008 | No |
| 2 | 103239278 | P2RX4 |  |  | 7912 | 1.149 | -0.2297 | Yes |
| 3 | 103225101 | GRIK3 |  |  | 8352 | 1.129 | -0.1289 | Yes |
| 4 | 103239451 | P2RX2 |  |  | 9019 | 1.085 | -0.0429 | Yes |
| 5 | 103240672 | GRIK2 |  |  | 12688 | 0.850 | -0.1160 | Yes |
| 6 | 103234753 | GRIK5 |  |  | 13041 | 0.831 | -0.0431 | Yes |
| 7 | 103219109 | GRIN3A |  |  | 13155 | 0.823 | 0.0396 | Yes |
| 8 | 103243074 | GRIN2C |  |  | 15051 | 0.705 | 0.0302 | Yes |
| 9 | 103233589 | GRIN3B |  |  | 16726 | 0.590 | 0.0183 | Yes |
| 10 | 103239277 | P2RX7 |  |  | 19171 | 0.376 | -0.0507 | Yes |
| 11 | 103239575 | GRIN1 |  |  | 19357 | 0.356 | -0.0211 | Yes |
| 12 | 103235472 | P2RX3 |  |  | 19814 | 0.301 | -0.0094 | Yes |
| 13 | 103234973 | GRIN2D |  |  | 20078 | 0.260 | 0.0066 | Yes |
| 14 | 103223600 | P2RX6 |  |  | 20242 | 0.235 | 0.0243 | Yes |
| 15 | 103248658 | GRIK4 |  |  | 20435 | 0.205 | 0.0376 | Yes |
| 16 | 103222965 | LOC103222965 |  |  | 20461 | 0.200 | 0.0578 | Yes |
| 17 | 103215880 | GRID1 |  |  | 20624 | 0.167 | 0.0684 | Yes |
| 18 | 103242171 | P2RX1 |  |  | 20894 | 0.102 | 0.0673 | Yes |
| 19 | 103242166 | P2RX5 |  |  | 21271 | 0.000 | 0.0505 | Yes |
| 20 | 119620174 | LOC119620174 |  |  | 22071 | 0.000 | 0.0148 | Yes |
Table: GSEA details
[plain text format]

  

Fig 2: EXCITATORY\_EXTRACELLULAR\_LIGAND\_GATED\_ION\_CHANNEL\_ACTIVITY(GO:0005231)
  
Blue-Pink O' Gram in the Space of the Analyzed GeneSet

  

Fig 3: EXCITATORY\_EXTRACELLULAR\_LIGAND\_GATED\_ION\_CHANNEL\_ACTIVITY(GO:0005231): Random ES distribution
  
Gene set null distribution of ES for
 **EXCITATORY\_EXTRACELLULAR\_LIGAND\_GATED\_ION\_CHANNEL\_ACTIVITY(GO:0005231)**
